# 

**Published:** 1915-09-11

**Authors:** 


### Colliery Districts and Cardiff Hospital.

Meetings to promote a more helpful interest in the affairs of the King Edward VII.'s Hospital, Cardiff, have recently been held, on successive Sunday afternoons, at the mining villages of Senghenydd and Abertridwr, the former so widely known as the place of the great colliery disaster of 1913. The meetings were addressed on each occasion by Mr. Leonard D. Rea, the secretary and general superintendent of the hospital, and Mr. Rowland Evans, J.P., the secretary of the Cardiff and District Society, who made well-reasoned and powerful appeals, which were received with great attention and with much sympathy, each meeting resulting in the adoption of a resolution pledging those present to contribute a penny per week each towards the funds of the Cardiff and District Hospital Society. This Society is the working-men's organisation for supporting the hospital, and also, it is confidently hoped, for erecting and maintaining convalescent homes to be used in connection with the hospital. Mr. Rea and Mr. Evans emphasised the value of organisation, and the meetings made it clear that if the needs and claims of the hospital are explained to the workers their response will be ready and generous. The general adoption of the penny a week scheme throughout the coalfield would ensure to the Cardiff Hospital freedom from financial embarrassment, and provide resources for extended usefulness, as the system would have the effect of increasing the colliers' aggregate contributions tenfold.

### News Items.

The Queen has sent a basket of grapes from Windsor to the Royal Hospital for Incurables, Putney Heath, of which institution Her Majesty is Patron.

The military section of the Metropolitan Hospital will be open to the general public for inspection on Saturday afternoon, September 11.

The Dean of St. George's Hospital announces that he has every reason to believe that the hospital will remain at Hyde Park Corner for many years to come.

Rates of Subscription : Twelve months, 6/6 ; Six months, 4/- ; Three months, 2/-, post free to any address in the United Kingdom. Abroad, Twelve months, 8/8; Six months, 5/-; Three months, 2/6, post free.—Address The Subscription Dept., "The Hospital," The Hospital Building, 28/29 Southampton Street, Strand, London, W.C.

By Sir ARBUTHNOT LANE, Bart., M.S.

**OPERATIVE TREATMENT OF FRACTURES.**

*SECOND EDITION. With* 226 *Illustrations.* 10s.

THE MEDICAL PUBLISHING COMPANY, LTD., 23 Bartholomew Close, E.C.

THE OPERATIVE TREATMENT OF
**CHRONIC INTESTINAL STASIS.**

With additional Chapters by
Drs. JAMES MACKENZIE, JORDAN and MUTCH.
*THIRD EDITION. With* 102 *Illustrations.* 10*s.* 6*d. net.*

J. NISBET & CO., LTD., 22 Berners Street, W.

**CLEFT PALATE AND HARE-LIP.**

With additional Chapters by
Mr. CORTLANDT MACMAHON and Mr. WARWICK JAMES.
*THIRD EDITION. With* 58 *Illustrations.* 10*s.*

THE MEDICAL PUBLISHING COMPANY, LTD., 23 Bartholomew Close, E.C.

A LIST of the NEW PUBLICATIONS of THE SCIENTIFIC PRESS, LIMITED, will be sent post free to any member of the Medical Profession on receipt of post card.

28 & 29 SOUTHAMPTON STREET, STRAND, LONDON, W.C.

